# Defining the Microglia Response during the Time Course of Chronic Neurodegeneration

**DOI:** 10.1128/JVI.02613-15

**Published:** 2016-02-26

**Authors:** James E. Vincenti, Lita Murphy, Kathleen Grabert, Barry W. McColl, Enrico Cancellotti, Tom C. Freeman, Jean C. Manson

**Affiliations:** The Roslin Institute and Royal (Dick) School of Veterinary Studies, University of Edinburgh, Easter Bush, Midlothian, United Kingdom

## Abstract

Inflammation has been proposed as a major component of neurodegenerative diseases, although the precise role it plays has yet to be defined. We examined the role of key contributors to this inflammatory process, microglia, the major resident immune cell population of the brain, in a prion disease model of chronic neurodegeneration. Initially, we performed an extensive reanalysis of a large study of prion disease, where the transcriptome of mouse brains had been monitored throughout the time course of disease. Our analysis has provided a detailed classification of the disease-associated genes based on cell type of origin and gene function. This revealed that the genes upregulated during disease, regardless of the strain of mouse or prion protein, are expressed predominantly by activated microglia. In order to study the microglia contribution more specifically, we established a mouse model of prion disease in which the 79A murine prion strain was introduced by an intraperitoneal route into BALB/cJ^*Fms-EGFP/*−^ mice, which express enhanced green fluorescent protein under the control of the *c-fms* operon. Samples were taken at time points during disease progression, and histological analysis of the brain and transcriptional analysis of isolated microglia was carried out. The analysis of isolated microglia revealed a disease-specific, highly proinflammatory signature in addition to an upregulation of genes associated with metabolism and respiratory stress. This study strongly supports the growing recognition of the importance of microglia within the prion disease process and identifies the nature of the response through gene expression analysis of isolated microglia.

**IMPORTANCE** Inflammation has been proposed as a major component of neurodegenerative diseases. We have examined the role of key contributors to this inflammatory process, microglia, the major resident immune cell population of the brain, in a murine prion disease model of chronic neurodegeneration. Our study demonstrates that genes upregulated throughout the disease process are expressed predominantly by microglia. A disease-specific, highly proinflammatory signature was observed in addition to an upregulation of genes associated with metabolism and respiratory stress. This study strongly supports the growing recognition of the important contribution of microglia to a chronic neurodegenerative disease process.

## INTRODUCTION

Over several decades, the neuron has been subject to the majority of research into protein misfolding diseases, but it is now apparent that glial cells are important players in the neurodegenerative process. Many protein misfolding diseases, including Alzheimer's disease, Parkinson's disease, and prion diseases, demonstrate activation of glial cells in the brain during the course of disease alongside accumulation of misfolded protein, but the precise role of the glial cells in the disease process is not known (1–4). Transmission of prion agents to mice provides an excellent model for studying the timing of events during a chronic process of a neurodegeneration associated with a misfolded protein. The time of inoculation defines the starting point for the disease process, and highly reproducible characteristics of mouse-adapted prions include the accumulation of a misfolded host protein, gliosis, neuronal loss, the distribution of brain lesions, and the endpoint of terminal disease. Activation of glial cells, both astrocytes and microglia, has been extensively documented as an early event in the pathogenesis of protein misfolding diseases, occurring well before the onset of clinical disease (1, 5–7).

Microglia are the major resident immune cell in the brain and in steady-state are considered a heterogeneous population with density differences across brain regions (8). They display region-dependent functional signatures, which are enhanced further by age (9). Under normal conditions microglia adopt a “resting” phenotype, where they continually survey their immediate environment with extended processes (10).

After detection of a pathological insult or any disturbance to homeostasis, microglia adapt their phenotype from “resting” to “activated,” whereby they modify both morphology and biological function (10–12). Activated microglia have diverse functional phenotypes dependent on the nature of the stimuli that are not readily apparent from their morphology and include a much wider repertoire than the classically defined M1 and M2 phenotypes (13–16). It has also been proposed that microglia can readily switch from one phenotype to another (17–19) and are sensitive to peripheral immune system communication (20–22). It is also clear that a complicated interconnected network of central nervous system (CNS) cells contribute to the activated “profile” adopted by microglia with signaling from both astrocytes and neurons having particular impact (23–26).

The change of microglia from a resting to an activated state is one of the first pathological features of prion disease long before there is any evidence of neurodegeneration. Activated microglia are widely distributed in the brain and are thought to express low levels of inflammatory cytokines but high levels of transforming growth factor beta 1 (Tgfb1) and prostaglandin E2 (PGE2) (7, 27).

This study aims to investigate the role of microglia through detailed analysis of their morphology and gene expression during the course of prion disease, thereby providing new insights into the pathophysiology of neurodegenerative disease. We used a prion disease agent as a model of neurodegeneration and take an unbiased whole-genome expression analysis approach, which has allowed us to provide insight into the molecular processes central to microglia during the neurodegeneration and highlight how this may impact disease development. A strong myeloid association was attributed to disease-associated genes identified in our reanalysis, supporting the growing recognition of the importance of microglia within the disease process. To further clarify the microglial contribution, we isolated microglia from prion-infected mice and analyzed their gene expression profiles.

## MATERIALS AND METHODS

### Reanalysis of published data.

The Hwang data set (28) was downloaded from http://prion.systemsbiology.net. Quality control of these data was performed by Fios Genomics, Ltd. (Edinburgh, Scotland), using the ArrayQualityMetrics (29), and 32 microarrays were removed due to poor quality, leaving 386, both infected and uninfected, for reanalysis. The removal of arrays did not affect the overall balance of the data set with a mean average of 2.5 ± 0.08 (standard error [SE]) arrays per time point. Data normalization was performed using the robust multiarray average expression measure (30).

Initial inspection of the data showed that there were no age-related trends present in the control animal data, and these were not included in further analyses. Data from prion-infected animals were loaded into BioLayout Express^3D^ (31), and a Pearson correlation matrix was calculated, comparing the expression data from each probe set on the array against all other probe sets (*P*^2^/2 pairwise calculations, where *P* is the number of probe sets).

A threshold of *r* ≥ 0.75 was used, and the resultant correlation graph was visualized. To identify groups of coexpressed genes, the graph was clustered using the graph-based Markov clustering (MCL) algorithm (32) with the inflation value set at 2.2. The expression profile of each cluster was inspected, clusters of genes differentially expressed during disease were isolated, and individual gene profiles were examined. Those with an unconvincing profile, i.e., their expression was weak or unrelated to disease progression, were removed. This left a data set comprising 492 genes in which there was high degree of confidence that their expression was upregulated during disease.

### Determination of cell type of origin and function of disease-associated transcripts.

Cell origin was determined with reference to existing data sets. A data set was compiled from data derived from a number of published studies and included microglia, macrophage, and osteoclast myeloid populations (33, 34); purified neuronal populations derived from the cortex (cholecystokinin^+ve^, cholinergic, layer 5a, layer 5b, layer 6, and prepronociceptin^+ve^); the striatum (dopamine receptor subtype 1 medium spiny, and dopamine receptor subtype 2 medium spiny) and the cerebellum (basket, Golgi, Purkinje, stellate, unipolar brush); and astrocytes, Bergman glia, and oligodendrocyte populations (35, 36). Finally, data sets derived from macrophage cultures cultured with lipopolysaccharide (LPS) bacterial endotoxin (37) were included to allow for the identification of genes associated with activation of the innate immune system. After normalization of the data, the 492 genes demonstrating differential expression in response to prion disease were identified in the composite data set through matching of gene symbols and incorporated into an expression file. Within BioLayout Express^3D^ each gene could then be assessed for their expression in one or more of these cell types.

Gene ontology enrichment was determined by uploading the Affymetrix chip ID of the disease-associated genes to the online Ensembl Biomart data mining tool (http://ensembl.org/biomart ) using the Mus musculus genes data set (Ensembl Genes 66). Filters were applied restricting results to the Affymetrix 430 2.0 chip probe sets. To increase accuracy for correct selection of function, filters for gene ontology evidence code, domain and name were applied, with experimental evidence codes preferred.

### Animals and treatment.

Groups of male and female BALB/cJ^*Fms-EGFP/*−^ mice, expressing enhanced green fluorescent protein (EGFP) under the control of the *c-fms* operon (part of the *Csfr1* promoter) (38), were sex matched and housed under standard conditions in groups of three to five. Food and water access was *ad libitum*. All mouse experiments were reviewed and approved by the local ethical review committee and performed under license from the UK Home Office in accordance with the United Kingdom Animal (Scientific Procedures) Act 1988. Mice aged 16 weeks old were challenged by an intraperitoneal (i.p.) route with 0.02 ml of 1% (wt/vol) (in physiological saline) 79A-infected or normal brain material (NBr) for a control. At time points 35, 100, 150, and 200 days postinoculation (dpi) mice were sacrificed (9 per group for immunohistochemical analysis and 4 per group for microglial extraction). All remaining mice (12 and 8 per group, respectively) were assessed for clinical signs of prion disease from 150 dpi, and incubation times were calculated according to previously described protocols (39). These mice were sacrificed during terminal disease, or earlier if welfare required. Tissue sections from these mice were assessed for spongiform degeneration according to previously described procedures by a scientist blinded to experimental design (40).

### Tissue preparation and immunohistochemical analysis.

Brains were removed at the selected time points. Those to be used for immunohistochemistry were perfusion fixed with saline, followed by 4% paraformaldehyde (PFA; pH 7.4). Brain tissue was embedded in paraffin and cut into sections (6 μm). Antigen retrieval was performed in an autoclave at 121°C for 15 min in distilled H_2_O and then incubated in formic acid (98%) for 10 min at room temperature. Endogenous peroxidase was blocked with 1% H_2_O_2_ (Sigma-Aldrich) in methanol for 10 min. All sections were blocked with serum-free protein block (Dako) or normal goat serum prior to incubation with the primary antibody. Sections were immunostained with monoclonal antibody (MAb) 6H4 (Prionics) recognizing residues 143 to 151 of murine PrP (0.5 μg ml^−1^) (41). Negative control slides were treated overnight with mouse immunoglobulin control (Invitrogen). Antibody binding was detected with Vector ABC kit (Vector Laboratories) and visualized with 3,3,-diaminobenzidine chromogen. All sections were counterstained with hematoxylin.

Brains for microglia morphology assessment were removed and immersed in 4% PFA for 24 h and then rinsed in Hanks balanced salt solution (HBSS) before incubation for a further 24 h in 20% sucrose solution at 4°C. Tissues were rinsed with HBSS and snap-frozen in isopentane at −40°C. Brains for microglial extraction were immersed in cold HBSS prior to processing (see “Microglial isolation procedure” below).

### Quantification of microglia morphology/phenotype.

Frozen brain tissue was sectioned at 25 μm on a freezing block microtome, and sequential sections 300 μm apart were taken for analysis. Quantification of microglia activation status was established on cellular aggregation and morphology observed in BALBcJ^*Fms-EGFP/*−^ sections based on the average number of microglia per 0.05 mm^2^. Images for cell quantification were captured as a 50 optical slice z-stack at ×10 magnification (Zeiss Plan-Neofluar 10×/0.30 objective) and compiled into a composite image using ImageJ software 1.48a. Quantification of the EGFP cell number was performed using particle analysis within ImageJ. Microglia radius was performed on ×10 z-stack compiled (reporting z-stacks) images taken at ×40 magnification (Zeiss Plan-Neofluar ×40/1.30 objective) captured from three standard locations within four brain regions: the dentate gyrus, cerebellum, medulla, and thalamus. There was a minimum of three mice per group, and additional images were recorded on adjacent sections if the total number of EGFP expressing microglia was below 50. Euclidean distance mapping was utilized to quantify changes in morphology and was performed using the “region of interest” function within ImageJ.

### Microglial isolation procedure.

Brains harvested for microglial extraction were placed in cold HBSS and diced before processing immediately. Brains were dissociated using a GentleMACS dissociator (Miltenyi Biotec) and a neural tissue dissociation kit P (Miltenyi Biotec). The final cell pellet was resuspended in 16 ml of 35% Isotonic Percoll, split between two 15-ml tubes, and carefully overlaid with 5 ml of ice-cold 0.1% diethyl pyrocarbonate (DEPC)-treated HBSS. The resulting Percoll gradient was centrifuged at 400 × *g* for 45 min at 4°C. The pellets were then suspended and recombined into a final volume of 5 ml ice cold 0.1% DEPC-treated HBSS. Cells were pelleted at 400 × *g* for 5 min at 4°C using no brake, resuspended in 90 μl of ice-cold MACS buffer (Miltenyi Biotec) and 10 μl of CD11b (microglia) microbeads (Miltenyi Biotec), and incubated at 4°C for 15 min with gentle rotation. After incubation with microbeads, the cell suspension was washed in 1 ml of ice-cold MACs buffer at 300 × *g* for 5 min at 4°C and then resuspended in 500 μl of ice-cold MACs buffer. Cells were passed through magnetized LS columns (Miltenyi Biotec) according to the manufacturer's protocol.

### Verification of microglial purity.

A subset of isolated cells predicted to be microglia were stained with phycoerythrin (PE) anti-mouse CD11b (Cambridge Bioscience) and allophycocyanin (APC) anti-mouse CD45 (Cambridge Bioscience). Isotype controls were prepared using PE-rat IgG2b (Cambridge Bioscience) and APC-rat IgG2a (Cambridge Bioscience), and a subset of unstained cells served both as a negative control and a verification of the correct BALB/cJ^*Fms-EGFP/*−^ genotype. Cell viability was determined using SYTOX Blue dead cell stain (Thermo Fisher Scientific). All cell samples were analyzed on a BD FACSAria IIIu 4-laser/11 detector cell sorter running BD FACSDiva software (BD Biosciences). Subsequent analysis of fluorescence-activated cell sorting (FACS) data was also performed using Summit v4.3 software (Dako/Beckham Coulter).

### Microarray analysis of isolated microglia.

Isolated microglia cells were treated with TRIzol Reagent (Life Technologies) according to the manufacturer's protocol. Total RNA quality was checked on an Agilent 2100 Bioanalyzer. RNA samples with RIN value of >7.0 were passed as suitable for analysis, and two representative samples at each time point for control and disease were taken forward for analysis. RNA processing was handled by Ark Genomics [The Roslin Institute and R(D)SVS]. RNA was converted to amplified double-stranded cDNA containing biotin using a NuGen Ovation picoSL WTA labeling kit (NuGen). The cDNA samples were hybridized to Affymetrix Mouse Gene 1.1 arrays on a GeneTitan instrument (Affymetrix). Data were quality controlled, RNA normalized, and subjected to network analysis as described above.

### Microarray data accession number.

The microarray data sets supporting the results in this article are available in the NCBI GEO repository under accession number GSE72039 .

## RESULTS

### The neurodegenerative disease process is associated with an inflammatory response which is microglial in origin.

**FIG 1:**
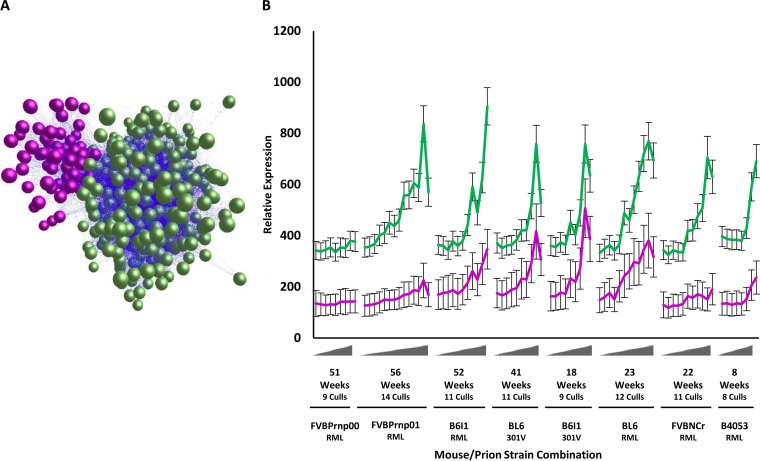
BioLayout Express^3D^-generated transcript -to-transcript network graph of selected genes of interest. (A) A list of 492 genes of interest with an expression pattern indicative of disease association is organized into two main clusters within BioLayout Express^3D^ by MCL. The green cluster comprising 410 nodes was joined by 29,339 edges, indicating a high degree of coexpression between genes. The smaller purple cluster comprised 67 nodes and 1,453 edges. (B) The disease-associated gene expression signatures of both clusters, displayed as a mean expression profile for each strain, revealed an upregulation at ca. 50% of the incubation period. The profile was similar for all genes in all mouse-prion combinations. The smaller purple cluster was expressed highest in BL6 strains, resulting in the formation of a separate cluster. Error bars indicate ± the SE. Gray triangles on the *x* axis indicate the incubation period between the point of inoculation and culling, with some mouse/TSE strains leading to pathology and death faster than others.

Initially, we performed a reanalysis of the data produced by Hwang et al. (28): a transcriptomics analysis of brains of multiple strains of mice infected with different prion strains sampled at various stages of disease progression. These analyses were performed with a view to identifying genes associated with neurodegenerative disease progression. The Hwang data from diseased animals were analyzed within BioLayout Express^3D^. A correlation graph was generated using a Pearson threshold of *r* ≥ 0.75, consisting of 21,550 nodes with 1,253,332 edges (Fig. 1A). Clustering with MCL yielded 416 clusters. Each cluster represented genes that share a high degree of coexpression. The expression profile of the majority of the clusters revealed they had an expression profile that was not linked to the disease process. Two major clusters of genes did however exhibit an expression profile that increased with disease progression in all animal-prion strain combinations (Fig. 1B). The largest of these clusters comprised 377 genes and a second contained 115 genes that were notable for their increased activation in C57BL/6 models (Fig. 1B). Following manual inspection of all individual profiles, a total of 492 genes associated with prion disease development were identified (see Table S1 in the supplemental material). All genes in each cluster followed a similar expression profile with an increase in expression starting at approximately halfway through the incubation period.

**FIG 2:**
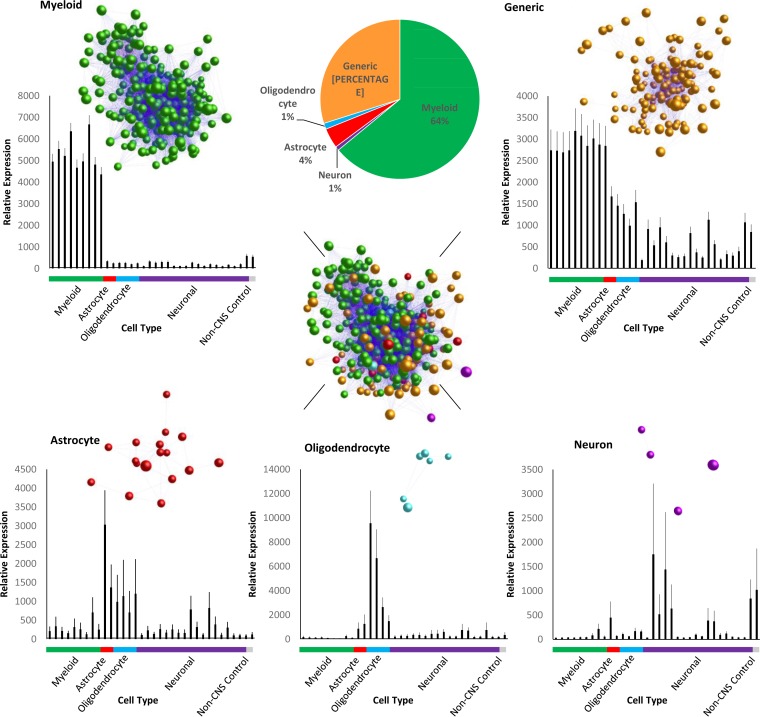
Cross reference of the 492 disease-associated genes with conormalized external data sets within BioLayout Express^3D^. Within BioLayout Express^3D^, each gene of interest was classed and colored as a specific cell type. Note how the previously determined MCL clusters are both dominated by myeloid-derived genes (green). Genes associated with myeloid were divided into two groups based on sole association with myeloid cell types or in which sole origin could not be determined. Genes with a sole myeloid origin comprised 318 genes or 64% of the gene set. A total of 146 genes were associated with multiple cell types found within the CNS. Here, a myeloid component was still observed as strongly associated with the group. Genes assigned to astrocytes, oligodendrocytes, and neurons were each represented by <20 genes.

Once disease association was determined, we next attempted to identify the cellular origin for each of the 492 differentially expressed genes in question. This was done by examining the expression of the disease-associated genes in the context of a panel of isolated cell populations. Data sets were sourced from the GNFv3 cell atlas (33, 34), RNA TRAP (35, 36), and serial macrophage cultures subjected to LPS (37). This revealed that 315 of the 492 differentially expressed genes were solely or at least predominantly expressed by myeloid populations, thereby indicating the majority were likely expressed by microglia within the brain. In contrast, 147 of the genes were expressed by multiple cell types, while only 30 were found to be specific to astrocytes, oligodendrocytes, and neurons collectively (Fig. 2). The original study by Hwang et al. (28) identified 333 differentially expressed genes. By overlaying these 333 genes onto our chosen external data sets within BioLayout Express^3D^ it was found that 158 of the 333 genes were attributed to a myeloid origin. A further 18 were attributed to nonmyeloid cell types. The remaining genes were classed as generic, implying the origin could be any cell within the brain and as such do not rule out a microglial component.

### Histological analysis of microglial activation and PrP deposition.

After the identification of the predominantly myeloid origin of the prion disease signature, we chose next to confirm this observation by performing an analysis of microglia isolated from diseased brains. Our aim was to verify these findings and to obtain a more detailed analysis of the activation of microglia during disease. To do this, we chose a mouse-passaged prion agent, 79A, inoculated into BALB/cJ^*Fms-EGFP/*−^ mice by an intraperitoneal route with 0.02 ml of 1% (wt/vol) 79A brain homogenate as our model.

Clinical disease onset occurred 198.5 ± 1.0 (SE) dpi with signs, including lethargy, hair unkempt/loss, and hunching, all reported. Terminal disease occurred 229 ± 3.6 dpi. Pathological analysis of the vacuolation in the brain of terminal animals (*n* = 6) confirmed clinical disease and indicated that vacuolation was widespread by terminal stage of disease presenting as typical for the 79A prion strain (42, 43).

**FIG 3:**
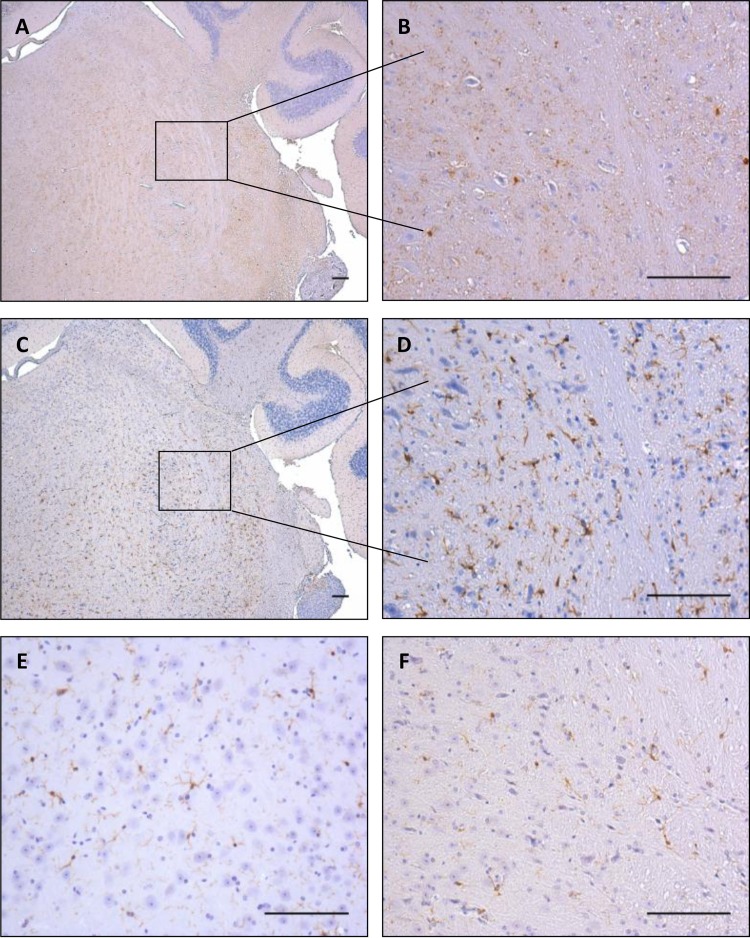
PrP deposition and microglia activation assessed by immunohistochemistry. Microglia activation was observed in the same areas as PrP deposition. (A and B) Earliest accumulation of PrP (6H4) is at 150 dpi in the medulla. (C and D) Microglia can be seen accumulating in the same areas of deposition. (E and F) Normal microglia in the thalamus and medulla, respectively, of mice challenged with normal brain demonstrate a ramified appearance and greater separation at ∼50 μm. All images representative. Scale bars, 100 μm.

PrP deposition assessed by immunohistochemistry using the 6H4 antibody was first detected in the infected mice at 150 dpi and restricted to the medulla (Fig. 3A and B). PrP assessment at the terminal stage of disease identified heavy accumulation of fine punctate particles throughout the majority of the brain, strongest in the thalamus and extending into the medulla. To a lesser extent, deposition was also observed within the hippocampus, but it was only occasionally found within the cortex. This is the deposition pattern typically associated with 79A disease progression (42, 44). Microglial activation was observed in the same areas as PrP deposition at 150 dpi (Fig. 3C and D). Microglia in the NBr-inoculated controls demonstrated a ramified appearance and greater microglia separation at ∼50 μm (Fig. 3E and F).

**FIG 4:**
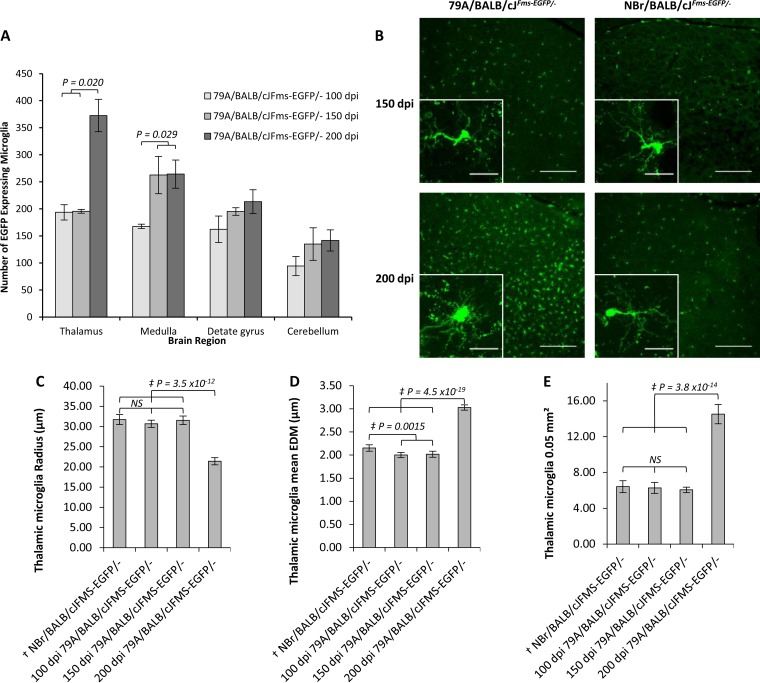
Software-determined quantification of EGFP-expressing microglia in 79A-infected BALB/cJ^*Fms-EGFP/*−^ mice. (A) Quantification of regional microglia cell number in BALB/cJ^*Fm*^[supi]s-EGFP/− mice following i.p. challenge with 79A at 100, 150, and 200 dpi. EGFP-expressing cells were counting using ImageJ particle analysis function on ×10 magnification, 25-μm z-stack compiled images each comprising 50 optical slices. Microglia density increases in the medulla by ∼50% at 150 dpi, whereupon the numbers remain constant in this region as PrP deposition spreads anteriorly. By 200 dpi, the microglia density in the thalamus has increased by ∼100%. (B) EGFP-expressing microglia in the thalamus of BALB/cJ^*Fms-EGFP/*−^ mice following i.p. challenge with 79A at 150 and 200 dpi. No difference in the number of EGFP-expressing cells was observed in the thalamus until 200 dpi, when a concentration of reactive microglia spaced <25 μm apart was observed. Before 200 dpi, microglia were observed in all animals as spaced at 50 to 100 μm and adopt a normal ramified morphology. Scale bars, 200 μm. Inset scale bars, 20 μm. (C) At 200 dpi, microglia present with an engorged central body and shortened processes conferring a significant reduction in radius. (D) Euclidean distance mapping affords a highly sensitive quantification of cell complexity encompassing both cell body size and process branching. The reduction in cell radius at 200 dpi is reflected in a mean Euclidean distance increase of 1 μm. Distance mapping also detailed a slightly less complex cell type in the NBr animals. (E) High-resolution image analysis of microglia density per 0.05 mm^2^ in the thalamus at 200 dpi revealed an increase of ∼100%. †, Comprises mean for all NBr-inoculated BALB/cJ^*Fms-EGFP/*−^ mice at all serial investigation time points. ‡, Mean statistical value determined using a *t* test, assuming variances determined by *f* test. NS, not significant. In panels A, C, D, and E, the error bars indicate ± the SE.

Microglia were identified during the course of disease using EGFP expression, and a quantitative analysis was performed on their density and radius, as a measurement of morphological changes typically associated with the activation of microglia. Upon comparison to animals that had been inoculated with uninfected NBr homogenate, we observed at 150 dpi an approximate 50% (*P* = 0.029) increase in microglial cell number per 0.05 mm^2^ within the medullas of 79A-infected mice (Fig. 4A). Similarly, at 200 dpi an increase of microglia of ca. 50% (*P* = 0.02) was observed within the thalamus. The intercellular distance of microglial in control and unaffected regions was approximately 70 to 100 μm, while within affected regions this was reduced to ∼25 μm (Fig. 4B). Cellular microglial activation was also defined by a marked increase in the diameter of the central body, while there is a reduction in the length and number of processes projecting from it (45). An average length of approximately 30 μm was observed for thalamic microglia at 100 days, while at 200 dpi this is reduced to an average of 20 μm, indicating morphology associated with activation. The reduction in radius is matched with an increase in Euclidean distance by 1 μm, similar to that seen in the microglia in the thalamus, and indicative of shorter thicker processes and a larger central body (Fig. 4C to E). Thus, the pathological analysis confirmed that microglial activation and PrP disease associated protein deposition occurs by 150 dpi in restricted regions of the brain, and during the course of disease both extend into multiple brain regions. There was no evidence of either PrP deposition or microglial activation at 100 days in this model.

### Microglial activation profile.

**FIG 5:**
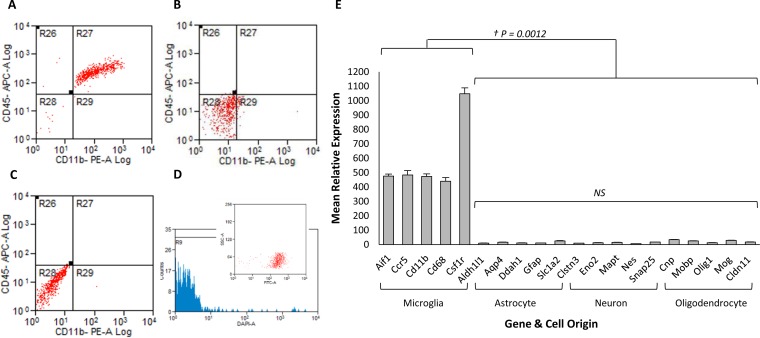
The purity of isolated microglia was confirmed to be high, and extracted RNA was confirmed to be of workable quality. (A) FACS sample analysis of CD11b microbead-purified microglia stained with, and positive for, CD11b-PE and CD45-APC demonstrate a high purity. (B and C) An isotope control (B) and a negative control (C) show no nonspecific binding or autofluorescence, respectively. (D) Cell viability was confirmed using SYTOX live-dead stain. The inset shows isolated cells are EGFP positive. (E) Plot of the mean expression profile of 20 genes known to be expressed in a cell-specific manner. The first five are known microglial expressed genes, and the remainder are expressed in other brain cell types. This demonstrates that the expression of nonmicroglia genes in isolated microglial populations is negligible, suggesting a relatively pure microglial population. Error bars indicate ± the SE.

Microglia were isolated at 35, 100, 150, and 200 dpi from 79A-inoculated and control animals. Isolated cells were stained with CD11b and CD45 fluorochrome-conjugated antibodies and sorted by FACS to confirm purity (Fig. 5). Adult microglia are typically shown as CD11b^High^ and CD45^Low^ (46), and the lower-than-expected CD11b forward and side scatter may be attributed to competition for available antigen between the CD11b microbeads and CD11b-PE marker. The number of CD45^high^ cells, indicative of impurities in the cell isolation process by monocyte contamination, was negligible. Nonspecific binding or autofluorescence was not observed. Cell viability was confirmed to be 97% ± 0.43% (SE). Microglia purity was further confirmed from the expression profile of 20 cell-specific genes representing the main cell groups found within the brain (Fig. 5E). The presence of CD11b^positive^ circulating or inflammatory monocytes was confirmed to be absent, as evidenced by the negligible expression of *Ly6c* or *Ccr2*.

The process of isolation did not appear to adversely affect the microglia disease signature. There was a clear difference between expression profiles of microglia isolated from diseased mice and those collected from uninfected controls. Of note was the lack of increased expression of metabolism genes that may be expected if cells were unduly stressed during the isolation process. Staining with SYTOX Blue also confirmed that cells from both infected and control animals were viable prior to RNA isolation. Additionally, on a bright-field microscope, isolated microglia presented with a rounded refractive appearance indicative of healthy viable cells.

**FIG 6:**
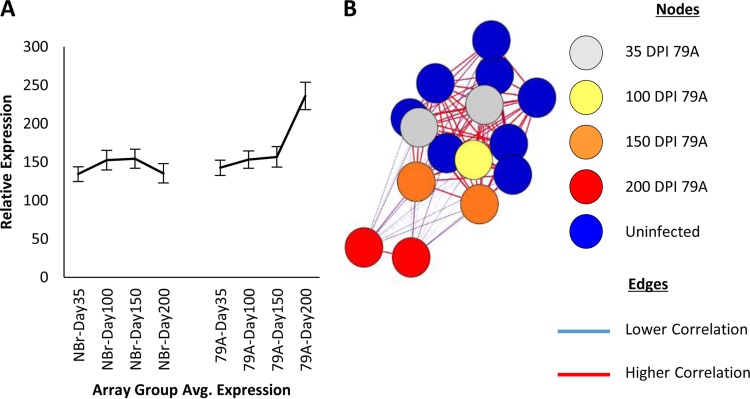
BioLayout Express^3D^ analysis of isolated microglia gene expression. (A) Average expression profile of the two large clusters comprised of 741 genes that demonstrate a differential expression in response to disease. All genes yielded an increase in expression with an upregulation at 200 dpi in 79A-infected mice. Error bars indicate ± the SE. (B) Global microarray sample-to-sample transposed BioLayout Express^3D^ graph of the 741 identified genes of interest. Prion-infected and uninfected pre-200 dpi arrays are highly correlated and organized into one component. Displaying high intercorrelation but lower correlation with the rest of the population are the arrays for the 200-dpi-infected group. Note that the nodes have been colored only for clarity and are not indicative of MCL clustering.

RNA was extracted and microarray analysis was performed. After this, the patterns of gene expression were analyzed within BioLayout Express^3D^. The expression profile of each cluster was individually checked to ensure familiarity with the data set, and those with a disease associated signature selected. This resulted in 741 genes that demonstrated an increase in expression predominantly at 200 dpi. The 741 genes were also organized into two large clusters which shared a very similar gene expression profile with a clear increase in expression profile (shown averaged in Fig. 6A). Animals inoculated with NBr showed no significant change in expression throughout the corresponding period. Using the 741 genes of interest, a sample-to-sample (array) level graph within BioLayout Express^3D^ was generated and confirmed the arrays (NCBI GEO accession number GSE72039 ) from the 200-dpi time point had less correlation with the rest of the samples (Fig. 6B).

### Gene enrichment analysis.

**FIG 7:**
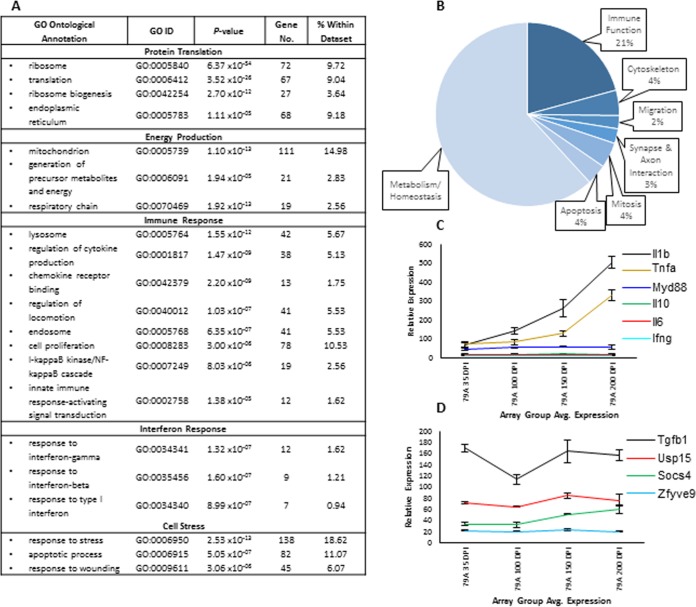
Ontological analysis of the microglia activation signature within the 741 genes of interest. (A) GO enrichment terms determined from the identified 741 differentially expressed genes using FuncAssociate 2.0 revealed protein translation, respiration, cellular stress, and components of the myeloid immune system to be significantly represented. All terms have a *P* value of considerably less than 0.001. (B) Regulated disease-associated genes allocated by function. Using the Ensembl Biomart database the majority of the regulated genes were ascribed to metabolism and homeostasis. Genes associated with immune system, for which differentiation has been included, comprise only a fifth. This highlights the power of a signal cell isolation in correctly determining the association of metabolic genes with a specific cell type. (C) Expression of inflammatory cytokines and transcription factors associated with the regulation of activation phenotype of microglia. The strong increase in the expression of *Tnfa* and *Il1b*, but not cytokines associated with recruitment and escalation toward acquired immunity, implies a disease-specific signature. (D) Nominal and unchanged expression of *Tgfb1* is matched by a lack of expression of downstream transcripts mediated by Tgfb1 activity. In panels C and D, error bars indicate ± the SE.

Enrichment analysis of the disease-associated microglial genes using the FuncAssociate 2.0 database (47) confirmed the enrichment (*P* < 0.001) of the following functional gene descriptions: translation, energy production, immune response, interferon response, and cell stress (Fig. 7A). Immunological response comprised the single largest category in respect to total gene number. The signature included transcripts associated with proteolysis, NF-κB-mediated cytokine cascades, and innate immunity. The GO enrichment functional groups of mitochondria, ribosome, cell stress, apoptotic process, and proliferation confirmed the presence of a significant metabolic signature associated with these genes.

Gene ontology was performed for each gene using the data made available on the Ensembl Biomart database to allow for functional associations to be determined (Fig. 7B). Just under two-thirds of the 741 identified differentially expressed genes were attributed to metabolism and the maintenance of homeostasis. The correct determination of differentially expressed metabolic genes to a specific cell type is only possible through the type of isolated cell type analysis presented here. Metabolic genes are typically expressed by all tissue cells types, making identification of the cellular origin from a mixed cell population impossible. Genes that were related to the immune response comprised just under a quarter of the total. The 6% of genes associated with cytoskeletal changes and migration were classed into their own groups, respectively, and included genes associated with membrane reshuffling. This was to be expected as microglia are known to be highly motile in the healthy brain environment (10). The increased expression of cytokines IL1, TNFa, and Csf1, but not IL6 or IL10, suggests that the response by microglia is lacking in the full spectrum of cytokines expected from a classical form of activation via the myeloid differentiation primary response 88 (Myd88)-dependent pathway (48). Pathway analysis of this data set within the Reactome database (49) revealed many of the elements of the Myd88-independent pathway were represented by the differentially expressed genes within this study. This is supported by the lack of differential expression of *Myd88* (Fig. 7C). In addition, *Tgfb1* was not found to be differentially expressed by microglia during the disease process (Fig. 7D). Transcripts associated with Tgfb1 signaling, including Smad anchor for receptor activation (*Zfyve9*), suppressor-of-cytokine signaling 3, 4, and 5 (*Socs3-5*), and ubiquitin-specific peptidase 15 (*Usp15*), were also absent.

**FIG 8:**
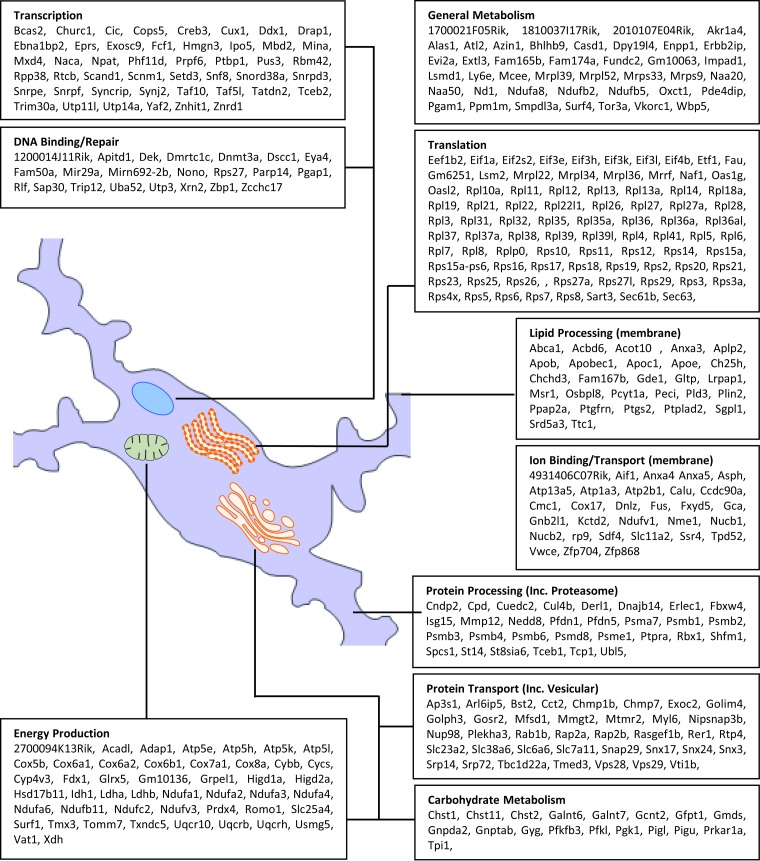
Genes of interest associated with metabolism and homeostasis. A considerable number of genes with an increase in expression are associated with protein translation and processing. The increased metabolic load is reflected in the increase in expression of genes associated with energy production.

Determination of the subcellular component for each gene considered to be associated with metabolism/homeostasis was performed from data obtained from the Ensembl Biomart database. This enabled the location of many genes to be plotted onto a cellular map and further organized by function (Fig. 8). The identified cellular components included a significant increase in expression of genes associated with ribosomes within the rough endoplasmic reticulum and cytoplasm. Indeed, the bulk of the metabolic genes were associated with the ribosomes, thereby implying an increase in ribosome numbers and/or ribosome turnover or an increase in protein synthesis. Also present was a significant concentration of genes associated with proteolysis, including proteasome-based ubiquitination.

Consistent with the increase in metabolic load were a considerable number of genes associated with oxidative phosphorylation and energy production in mitochondria, including subunits for cytochrome *c* oxidase, NADH dehydrogenase, and lactate dehydrogenases; the latter are associated with the breakdown of increased levels of lactate in situations of respiratory stress (50). Also observed were DNA repair processes, including the expression of poly(ADP-ribosyl)ation-14 (*Parp14*), a potent transcriptional regulator and DNA damage-dependent nuclear protein (51, 52).

**FIG 9:**
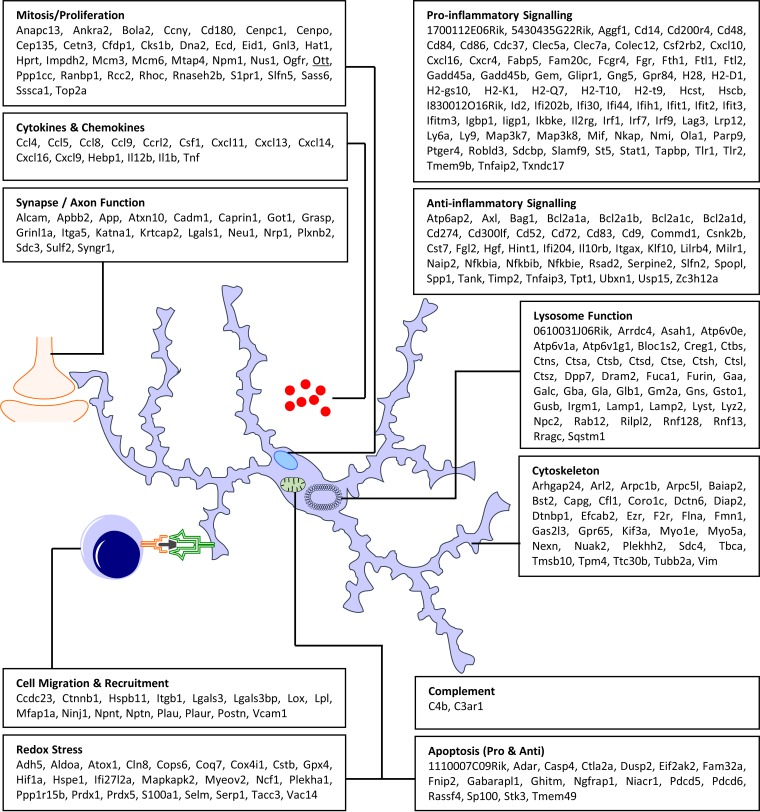
Genes of interest associated with immune activation and cell-to-cell signaling. Genes have been grouped by both function and cellular location. The signature is one of robust proinflammatory innate immune activation.

The association of the identified genes of interest with a specific function outside metabolism/homeostasis was plotted onto a cellular map using ontology data obtained from the Ensembl Biomart database. This enabled the location of each gene to be determined and further organized by function (Fig. 9). The overall expression profile from this set of genes is one of robust proinflammatory myeloid cell activation. The increased expression of lysosome-associated membrane protein, ATPase proton pumps, and numerous lysosomal enzymes, including cathepsins, histocompatibility subunits, and genes involved in membrane restructuring, strongly support antigen presentation and are a hallmark of classically activated innate immune cells. Increased expression of surface marker transcripts *Cd48*, *Cd86*, *Ccl8*, *Cxcl9*, *Cxcl13*, and *Tlr2* was also observed, and all are typically associated with a proinflammatory classical activation phenotype (16, 53–55).

## DISCUSSION

The data set generated by Hwang et al. (28) is uniquely placed among transcriptome data sets since it is the first to be fully comprehensive in terms of prion-related disease models, encompassing as it does multiple prion strains and host backgrounds. Our reanalysis of these data using a correlation network-based approach in combination with a cell origin classification system has given a unique, unbiased, and informative whole-genome approach. This allows identification not only of a core set of genes involved but also of cell types associated with the neurodegenerative disease process. We identified a further 299 disease-associated genes not reported in the original study by Hwang et al. (28) (see Table S1 in the supplemental material). The original analysis focused on defining pathways associated with disease progression, which speculated a prominent neuronal contribution to the disease signature. However, our reanalysis identified a large proportion of those previously identified genes to be of a myeloid origin with a strong myeloid association being attributed to 315 of the 492 disease-associated genes. This supports the growing recognition of the importance of microglia within the disease process. To further clarify microglial contribution, we isolated microglia from prion infected mice and analyzed their gene expression profiles.

Experimental differences between the present study and that of Hwang et al. (28), including route of infection, single cell type analysis, and RNA amplification, potentially limit the ability to directly compare the results of the two studies. Having said this, 107 genes were seen to be upregulated during disease progression in both studies and were primarily associated with an innate immune response (see Fig. S1 in the supplemental material). We adopted an intraperitoneal route of infection as a “more natural” route of infection rather than the more commonly used intracranial route to ensure that microglial activation was the result of a response to initial infection entering the CNS environment. With an intracranial route of infection, the microglial response may be complicated by the injection procedure, resulting in what has been termed the “prepriming” of microglia (56-58). A peripheral route would also encapsulate any microglial response to systemic inflammation observed in prion disease following a peripheral route of infection (59) but not following an intracerebral route of infection (60). However, both studies arrive at the same conclusion: prion disease is associated with a chronic inflammatory response, with microglia being central to the disease process.

The increase in the levels of *Il1b*, *Tnfa*, and *Csf1* strongly portray the microglial activation profile as proinflammatory and not one of atypical downregulation or resolution of inflammation (61). The presence of a significant increase in transcripts involved with proteasome activity and major histocompatibility complex-mediated antigen presentation, combined with expression of *Cxcr3* ligand genes, offers a microglia activation state more akin to classically activated macrophages. That said, the lack of expression of *Infg*, *Il6*, and *Il33* by microglia, all well-defined proinflammatory cytokines (62–64), suggests an atypical inflammatory response. Also of note, and crucial to the maintenance of a chronic response to inflammatory cytokines, was that the expression of *Nfkb1* remained stable despite an increase in expression of Nfkb1 inhibitors (*Nfkbia*, *Nfkbib*, and *Nfkbie*), which have been shown to inhibit formation of NFKB1 at the transcription stages (65).

The inflammatory phenotype typically associated with prion disease has been shown to be remarkably anti-inflammatory and dominated by the anti-inflammatory growth factor Tgfb1 following injection by an intracerebral (66) or a hippocampal stereotactic (7, 61) route. Found in the healthy brain, Tgfb1 is a constitutively expressed protein intricately involved in microglia homeostasis (67–70). The lack of differential expression of *Tgfb1* within this data set suggests a lack of active Tgfb1-mediated signaling as a significant contributor to the disease response by microglia. There was also lack of significant increase in expression of *Usp15*, *Zfyve9*, or *Socs3-5*, indicating no increased translocation of SMAD2/3 proteins or mitogen-activated protein kinase signaling, which are core intracellular complexes of the TGFB1 signaling pathway (67, 71–73). Tgfb1 is required for the correct function of the blood-brain barrier and is itself unable to pass (74, 75). This therefore suggests that the increased expression of Tgfb1 noted in other studies is either attributable to the intracerebral inoculation or expressed by another group of cells from within the CNS.

Microglia are known to intricately interact with neurons (76–78), and numerous genes associated with axon elongation, synapse regulation, and neurotransmitter release were observed to increase in expression within the isolated microglia data set. This partners the expression of many axon and synapse genes with microglia and adds them to the growing body of evidence for microglial involvement in neuron regulation (79–83). It has been proposed that microglia kill prion-infected neurons in a manner dependent upon the presence and degree of fibrillarity of misfolded protein (84). This single cell data set supports the generation of a neurotoxic response from microglia with increased expression of *Il1b*, *Tnfa*, and caspase-4 (*Casp4*), indicating active processing within caspase-1-mediated inflammasomes (85–87). Other proinflammatory genes found within this data set, and reported to be neurotoxic, include matrix metalloproteinase 12 (*Mmp12*) (88) and prostaglandin-endoperoxide synthase 2 (*Ptgs2*) (89). The latter is known to be expressed in prion disease (90, 91) and is a target of nonsteroidal anti-inflammatory drugs used in clinical trials to treat neurodegenerative diseases by inhibiting prostaglandin synthesis (92).

Within this analysis microglia were shown to express a disease signature markedly more proinflammatory than that currently portrayed in the literature for prion disease and more akin to other protein-misfolding diseases, notably Alzheimer's disease, in which microglia are observed as expressing a repertoire of proinflammatory cytokines, including Tnfa, Il1b, and Il6 (93–95). The increased expression of cytokines Il1b, Tnfa, and Csf1, but not Il6 in this data set suggests an activation profile that is specific to prion disease and likely also unique to the *in vivo* environment since cocultures of microglia and neurons in the presence of PrP^106−126^ induces a stereotypic response with CD14-mediated detection of damaged neurons and increased expression of Il6 (96). This matches the stereotypic neurotoxic response observed in cocultures of neurons in the presence of LPS activated microglia (97).

Our study demonstrated that genes upregulated throughout the disease process are expressed predominantly by microglia. A disease-specific highly proinflammatory signature was observed, in addition to an upregulation of genes associated with metabolism and respiratory stress. These findings strongly support the growing recognition of the important contribution of microglia to a chronic neurodegenerative disease process. Protein misfolding diseases typically have a very long preclinical phase in which there is a steady and progressive increase in misfolded protein deposition, neuroinflammation, and synaptopathy as the disease progresses. Thus, an understanding of the contributors to this preclinical phase provides opportunities for devising early intervention strategies to limit the pathology before damage becomes irreversible.

## Supplementary Material

Supplemental material
