# Supplementary material for: Defining the Microglia Response during the Time Course of Chronic Neurodegeneration
Source: J Virol. 2016 Feb 26;90(6):3003–17. doi: 10.1128/JVI.02613-15 (PMC4810622; doi:10.1128/JVI.02613-15)
Supplement: Supplemental material [file supp_90_6_3003__index.html]

Defining the Microglia Response during the Time Course of Chronic Neurodegeneration — Supplemental material 

# Defining the Microglia Response during the Time Course of Chronic Neurodegeneration

## Supplemental material

- Supplemental file 1 -

  Table S1 (The 492 disease-associated genes found in the reanalysis, organized by cell type.)

  Fig. S1 (The 107 genes shared between the two analyses.)

  PDF, 98K
