# Supplementary material for: Defining the Microglia Response during the Time Course of Chronic Neurodegeneration
Source: J Virol. 2016 Feb 26;90(6):3003–17. doi: 10.1128/JVI.02613-15 (PMC4810622; doi:10.1128/JVI.02613-15)
Supplement: Supplemental material [file JVI.02613-15_zjv999091443so1.pdf]

*Supplementary table 1-The 492 disease associated genes found in this reanalysis organised by cell type.*

| Cell Type | Gene No. | Gene Names | Unique No. | Unique Genes to this Study | Hwang *et al.*, (2009) No. | Gene Names Found in the Hwang *et al.*, (2009) Dataset (Not Cell Type Association) |
|---|---|---|---|---|---|---|
| **Macrophage Only** | 315 | 4632428N05Rik, A830007P12Rik, AA467197, Abi3, Adcy7, Adfp, AF251705, Aif1, Alox5ap, Anxa2, Anxa3, Anxa4, Arhgdib, Arpc1b, Aspg, Axl, B2m, Bcl2a1a, Bcl3, Brd4, C1qa, C1qb, C1qc, C3, C3ar1, C4b, Capg, Casp1, Casp8, Ccl12, Ccl2, Ccl3, Ccl5, Ccl6, Ccl8, Ccl9, Ccr5, Cd14, Cd37, Cd44, Cd48, Cd52, Cd53, Cd68, Cd72, Cd74, Cd84, Cd86, Cebpa, Cebpb, Ch25h, Clec4a3, Clec7a, Clic1, Cln3, Cmtm3, Cmtm6, Cmtm7, Cp, Crispld2, Csf1, Csf1r, Csf2rb, Csf3r, Cst7, Cstb, Ctsa, Ctsb, Ctsc, Ctsd, Ctsh, Ctsl, Ctss, Ctsz, Cxcl10, Cxcl13, Cxcl16, Cyba, Cybb, Ddx58, Ddx60, Dock2, Dtx3l, Ebi3, Edem1, EG667823 (Trim5), Emr1, Epsti1, Fas, Fcer1g, Fcgr1, Fcgr2b, Fcgr3, Fcgr4, Fcrls, Fermt3, Fgd2, Ftl1, Ftl2, Fuca1, Fxyd5, Fyb, Galnt6, Gbp2, Gbp3, Ggta1, Glipr1, Gmfg, Gngt2, Gns, Gpnmb, Gpr183, Gpr34, Gpr65, Gpr84, Gpsm3, Grn, Gusb, H2-Ab1, H2-DMb2, H2-K1, H2-M3, H2-Q7, Havcr2, Hck, Hcls1, Hexa, Hexb, Hfe, Hmox1, Hpgd, Hsd3b7, Hvcn1, Icam1, Ifi204, Ifi205, Ifi27l2a, Ifi30, Ifi35, Ifi44, Ifi47, Ifih1, Ifit1, Ifit2, Ifit3, Ifitm3, Igtp, Iigp1, Ikzf1, Il10rb, Il13ra1, Inpp5d, Irf5, Irf7, Irf8, Irf9, Irgm1, Irgm2, Itgam, Itgax, Itgb2, Itpripl2, Kcnk6, Lag3, Lair1, Lamp2, Laptm5, Lat2, Lcp1, Lcp2, Lgals3bp, Lgals9, Lgmn, Lilrb4, Lpcat2, Lpxn, Lrrc33, Lst1, Ltbr, Ly9, Lyn, Mafb, Man2b1, Man2b2, Mdfic, Mmp12, Mpa2l, Ms4a6b, Ms4a6d, Ms4a7, Msn, Myd88, Myo1f, Naglu, Naip2, Naip5, Ncf1, Ncf2, Ncf4, Nckap1l, Nek6, Nfe2l2, Nlrc5, Oas1g, Oasl2, OTTMUSG00000000971, P2ry13, P2ry6, Parp12, Parp14, Parp3, Parp9, Phf20l1, Pld4, Plec1, Plek, Plp2, Plxnb2, Pon3, Pros1, Psmb8, Psmb9, Psme1, Psme2, Ptgds2, Ptplad2, Ptpn18, Ptpn6, Ptprc, Pycard, Pyhin1, Rab32, Rab3il1, Renbp, Rnase4, Rnaset2a, Rnf213, Rrad, Rsad2, Rtp4, S100a11, S100a4, S100a6, S1pr3, Samd9l, Samsn1, Sash3, Scpep1, Selplg, Sema4d, Sepx1, Sfpi1, Sgpl1, Sh3bp2, Shisa5, Siglec5, Siglech, Skap2, Slamf9, Slc11a1, Slc15a3, Slc29a3, Slc39a14, Slc43a3, Slc5a3, Slc7a7, Slfn2, Slfn8, Socs3, Spp1, Sqrdl, Srgn, Stat1, Stat3, Sulf2, Syngr2, Tagln2, Tap2, Tapbp, Tbxas1, Tcn2, Tgfb1, Tgfbr1, Tgfbr2, Tgif1, Tgm2, Thbs2, Tifa, Timp1, Timp2, Tln1, Tlr1, Tlr13, Tlr2, Tm4sf1, Tmem119, Tmem173, Tmem86a, Tnfaip2, Tnfaip8l2, Tnfrsf1a, Tor3a, Trem2, Trim25, Trim30, Trim34, Trim47, Trpt1, Tspo, Tyrobp, Ube1l, Unc93b1, Usp18, Vwa5a, Xaf1 | 182 | 4632428N05Rik, A830007P12Rik, AA467197, Abi3, Adfp, Anxa2, B2m, Bcl3, Brd4, C3ar1, Capg, Ccl12, Ccl2, Ccl5, Ccl6, Ccl8, Ccr5, Cd14, Cd37, Cd44, Cd72, Cebpb, Ch25h, Clec4a3, Cln3, Cmtm3, Cmtm7, Cp, Csf1, Csf2rb, Csf3r, Cstb, Ctsa, Ctsb, Ctsh, Ctsl, Ctsz, Cxcl13, Cxcl16, Ddx58, Ddx60, Ebi3, Epsti1, Fas, Fcgr1, Fcgr2b, Fcgr4, Fermt3, Fgd2, Ftl2, Fuca1, Fxyd5, Fyb, Galnt6, Glipr1, Gmfg, Gngt2, Gns, Gpr183, Gpr65, Gpr84, Gpsm3, Gusb, H2-Ab1, H2-M3, Havcr2, Hck, Hcls1, Hexa, Hmox1, Hvcn1, Icam1, Ifi204, Ifi205, Ifi35, Ifi44, Ifi47, Iigp1, Il10rb, Il13ra1, Irf5, Irf7, Itgam, Lag3, Lair1, Lamp2, Laptm5, Lat2, Lcp1, Lcp2, Lpcat2, Lrrc33, Lst1, Ltbr, Ly9, Lyn, Man2b1, Man2b2, Mmp12, Mpa2l, Ms4a7, Myd88, Myo1f, Naip2, Naip5, Ncf1, Ncf2, Ncf4, Nckap1l, Nek6, Nlrc5, OTTMUSG00000000971, Parp14, Parp3, Parp9, Plec1, Plek, Plp2, Plxnb2, Pon3, Psme1, Psme2, Ptpn18, Pyhin1, Rab32, Rab3il1, Rrad, Rsad2, S1pr3, Samd9l, Samsn1, Sash3, Scpep1, Sema4d, Sepx1, Sfpi1, Sh3bp2, Shisa5, Slamf9, Slc11a1, Slc15a3, Slc29a3, Slc43a3, Slc5a3, Slc7a7, Slfn2, Slfn8, Socs3, Spp1, Sqrdl, Stat1, Stat3, Sulf2, Syngr2, Tagln2, Tap2, Tapbp, Tbxas1, Tgfb1, Tgfbr1, Tgfbr2, Tgif1, Tgm2, Tifa, Timp1, Timp2, Tln1, Tlr1, Tlr13, Tm4sf1, Tmem173, Tmem86a, Tnfaip2, Tnfaip8l2, Tor3a, Trim25, Trim30, Trim34, Trim47, Tspo, Ube1l, Usp18 | 133 | Adcy7, AF251705, Aif1, Alox5ap, Anxa3, Anxa4, Arhgdib, Arpc1b, Aspg, Axl, Bcl2a1a, C1qa, C1qb, C1qc, C3, C4b, Casp1, Casp8, Ccl3, Ccl9, Cd48, Cd52, Cd53, Cd68, Cd74, Cd84, Cd86, Cebpa, Clec7a, Clic1, Cmtm6, Crispld2, Csf1r, Cst7, Ctsc, Ctsd, Ctss, Cxcl10, Cyba, Cybb, Dock2, Dtx3l, Edem1, EG667823 (Trim5), Emr1, Fcer1g, Fcgr3, Fcrls, Ftl1, Gbp2, Gbp3, Ggta1, Gpnmb, Gpr34, Grn, H2-DMb2, H2-K1, H2-Q7, Hexb, Hfe, Hpgd, Hsd3b7, Ifi27l2a, Ifi30, Ifih1, Ifit1, Ifit2, Ifit3, Ifitm3, Igtp, Ikzf1, Inpp5d, Irf8, Irf9, Irgm1, Irgm2, Itgax, Itgb2, Itpripl2, Kcnk6, Lgals3bp, Lgals9, Lgmn, Lilrb4, Lpxn, Mafb, Mdfic, Ms4a6b, Ms4a6d, Msn, Naglu, Nfe2l2, Oas1g, Oasl2, P2ry13, P2ry6, Parp12, Phf20l1, Pld4, Pros1, Psmb8, Psmb9, Ptgds2, Ptplad2, Ptpn6, Ptprc, Pycard, Renbp, Rnase4, Rnaset2a, Rnf213, Rtp4, S100a11, S100a4, S100a6, Selplg, Sgpl1, Siglec5, Siglech, Skap2, Slc39a14, Srgn, Tcn2, Thbs2, Tlr2, Tmem119, Tnfrsf1a, Trem2, Trpt1, Tyrobp, Unc93b1, Vwa5a, Xaf1, |
| **Macrophage & Neuron** | 7 | A2m, Cyb5r3, Glipr2, Gpx1, Lgals1, Lgals3, Olfml3 | 3 | Cyb5r3, Glipr2, Gpx1, | 4 | A2m, Lgals1, Lgals3, Olfml3, |
| **Astrocyte Only** | 19 | Abhd4, Aldh1l1, Aqp4, Casp12, Chi3l1, Cyp4f14, Decr1, Fgfrl1, Gal3st4, Gm967, Id3, Naprt1, Plcd4, Plscr2, Prelp, Prrx2, Rbp1, Slc25a18, Sox9 | 9 | Abhd4, Aldh1l1, Decr1, Fgfrl1, Gm967, Plcd4, Prelp, Prrx2, Rbp1, | 10 | Aqp4, Casp12, Chi3l1, Cyp4f14, Gal3st4, Id3, Naprt1, Plscr2, Slc25a18, Sox9, |
| **Neuron Only** | 4 | Aldh1l2, Hspb8, Sema3d, Serping1 | 1 | Sema3d, | 3 | Serping1, Hspb8, Aldh1l2, |
| **Astrocyte & Neuron** | 11 | Ccdc122, Fxyd1, Kcne1l, Lgi4, Lmcd1, Mlc1, S100a16, Slc14a1, Thbs3, Tsc22d4, Vwa1 | 8 | Ccdc122, Kcne1l, Lmcd1, Mlc1, S100a16, Slc14a1, Thbs3, Tsc22d4, | 3 | Fxyd1, Lgi4, Vwa1, |
| **Macrophage, Neuron and Astrocyte** | 27 | Cd81, Cotl1, Cyp4v3, Dbi, Ifi27l1, Igfbp2, Itgb5, Ltc4s, Mt1, Mt2, Necap2, Npc2, Oat, Pdlim4, Rab31, Rab7l1, Rhbdf1, Rhoc, S100a13, Sh3glb1, Tmbim1, Tmem176a, Tmem176b, Tspan4, Ucp2, Vamp8, Vim | 16 | Cd81, Cotl1, Cyp4v3, Ifi27l1, Igfbp2, Ltc4s, Necap2, Npc2, Oat, Rab31, Rhbdf1, Sh3glb1, Tmbim1, Tspan4, Vamp8, Vim, | 11 | Dbi, Itgb5, Mt1, Mt2, Pdlim4, Rab7l1, Rhoc, S100a13, Tmem176a, Tmem176b, Ucp2, |
| **Macrophage & Astrocyte** | 28 | CD9, Cd151, Clec5a, Cybrd1, Dhrs1, Efemp2, Eya4, Galnt4, Gfap, Gstm1, Klk8, Lpcat3, Ly86, Mtmr11, Npl, Nupr1, Pbxip1, Pdpn, Pnpla7, Prdx6, Retsat, S100a1, Sat1, Sh3pxd2b, Tlr3, Trf, Upp1, Vcam1, Zfp91 | 18 | Clec5a, Cybrd1, Efemp2, Gfap, Gstm1, Lpcat3, Ly86, Mtmr11, Nupr1, Pnpla7, Retsat, S100a1, Sat1, Sh3pxd2b, Tlr3, Upp1, Vcam1, Zfp91, | 10 | Cd151, Dhrs1, Eya4, Galnt4, Klk8, Npl, Pbxip1, Pdpn, Prdx6, Trf, |
| **Oligodendrocyte** | 8 | Tppp3, Tmem98, Pdlim2, Klk6, Galntl2, Dbndd2, Apod, Adamtsl4, | 6 | Tppp3, Tmem98, Pdlim2, Galntl2, Dbndd2, Adamtsl4, | 2 | Apod, Klk6 |
| **Generic** | 73 | Znrf2, Tax1bp3, Srebf1, Spsb1, Spata13, Slco2b1, Scrg1, Scamp2, Rgs10, Pik3ap1, Phyhd1, Palld, P2rx7, Osmr, Nat2, Mrps6, Lyz2, Lyz1, Lrrfip1, Litaf, Klhl6, Itpkb, Il33, Igsf6, Igf1, Ifitm2, Idh2, Hspb6, H2-DMb1, H2-Aa, Gpr37l1, Golm1, Gbp6, Gba, Fzd9, Fkbp7, Fes, Farp1, Fam129b, Fam102a, F13a1, Ephx1, Entpd1, Emp3, Emp1, Ecm1, E230029C05Rik, Dok1, Ddr1, Dap, D4Ertd22e, Cyth4, Cyp7b1, Cst3, Col16a1, Cnn3, Clu, Cklf, Cebpd, Cd9, Ccdc80, Capn3, Bmp2k, Bdh2, BC026585, Avp, Atp6v0e, Angpt1, Ampd3, Aga, Afp, 5430427O19Rik, 1700112E06Rik | 56 | Znrf2, Tax1bp3, Srebf1, Spsb1, Scamp2, Rgs10, Pik3ap1, Palld, P2rx7, Nat2, Mrps6, Lyz2, Litaf, Klhl6, Itpkb, Il33, Igsf6, Igf1, Idh2, H2-DMb1, H2-Aa, Gpr37l1, Golm1, Gbp6, Gba, Fzd9, Fkbp7, Fes, Farp1, Fam129b, Fam102a, F13a1, Ephx1, Entpd1, Emp3, Emp1, Ecm1, Dok1, Ddr1, Dap, D4Ertd22e, Cst3, Col16a1, Cnn3, Cklf, Ccdc80, Capn3, Bmp2k, Bdh2, BC026585, Avp, Atp6v0e, Aga, Afp, 5430427O19Rik, 1700112E06Rik | 17 | Spata13, Slco2b1, Scrg1, Phyhd1, Osmr, Lyz1, Lrrfip1, Ifitm2, Hspb6, E230029C05Rik, Cyth4, Cyp7b1, Clu, Cebpd, Cd9, Angpt1, Ampd3 |
| **Totals** | **492** | | **299** | | **193** | |

***Supplementary Figure 1***

| Shared Genes | GO Ontological Annotation |
|---|---|
| 1700112E06Rik, Aif1, Anxa3, Anxa4, Arpc1b, Atp6v0e, Axl, Bcl2a1a, C3ar1, C4b, Capg, Ccdc122, Ccl5, Ccl8, Ccl9, Cd14, Cd48, Cd52, Cd72, Cd84, Cd86, Cd9, Ch25h, Clec5a, Clec7a, Csf1, Cst7, Cstb, Ctsa, Ctsb, Ctsd, Ctsh, Ctsl, Ctsz, Cxcl10, Cxcl13, Cxcl16, Cybb, Cyp4v3, Eya4, Fcgr4, Ftl1, Ftl2, Fuca1, Fxyd5, Galnt6, Gba, Glipr1, Gns, Gpr65, Gpr84, Gusb, H2-K1, H2-Q7, Ifi204, Ifi27l2a, Ifi30, Ifi44, Ifih1, Ifit1, Ifit2, Ifit3, Ifitm3, Iigp1, Il10rb, Il13ra1, Irf7, Irf9, Irgm1, Itgax, Lag3, Lamp2, Lgals1, Lgals3, Lgals3bp, Lilrb4, Ly9, Lyz2, Mmp12, Naip2, Ncf1, Npc2, Oas1g, Oasl2, Parp14, Parp9, Plxnb2, Psme1, Ptplad2, Rhoc, Rsad2, Rtp4, S100a1, Sgpl1, Slamf9, Slfn2, Spp1, Stat1, Sulf2, Tapbp, Timp2, Tlr1, Tlr2, Tnfaip2, Tor3a, Vcam1, Vim | • Immune system process: $P = 2.43\text{x}10^{-32}$ (GO:0002376)<br>• innate immune response: $P = 3.26\text{x}10^{-19}$ (GO:0045087) |

***Genes shared by both analyses.***

107 genes are shared between the two analyses. These genes comprise 22% of the total number of genes identified in the whole brain total-RNA re-analysis and demonstrate an ontological annotation associated with an innate immune response. Differences in the type of analysis negate further conclusions drawn from this comparison.
